# Metamizole-Induced Agranulocytosis: A Case Report

**DOI:** 10.7759/cureus.94983

**Published:** 2025-10-20

**Authors:** Joana Cartucho, Ana Ruivo, Ines Bonito, Carla Fernandes, Maria do Rosário Ginga

**Affiliations:** 1 Internal Medicine, Unidade Local de Saúde do Arco Ribeirinho, Barreiro, PRT

**Keywords:** adverse-drug reaction, agranulocytosis, bone marrow suppression, metamizole, neutropenic fever

## Abstract

Metamizole, a widely used analgesic and antipyretic, has been associated with rare but potentially life-threatening hematological adverse reactions, such as agranulocytosis. This case report highlights the diagnostic complexity and clinical management of a patient who presented with neutropenic fever and concurrent multidrug-resistant infections, ultimately found to be related to metamizole-induced agranulocytosis. It underlines the importance of considering drug-induced causes in the differential diagnosis of agranulocytosis and stresses the relevance of early identification and prompt discontinuation of the causative agent.

## Introduction

Metamizole (dipyrone) is a pyrazolone derivative with analgesic, antipyretic, and spasmolytic properties. Its use has been associated with agranulocytosis, a rare but potentially life-threatening adverse reaction characterized by a marked reduction in neutrophil count (<0.5×10⁹/L), predisposing to severe infections [1,2].

Despite these risks, metamizole remains widely available in countries such as Portugal, Spain, and Germany, where it is frequently used for the treatment of pain and fever [2,3]. In contrast, it has been withdrawn or restricted in others, including the United States, United Kingdom, and several Nordic countries, due to concerns over hematologic toxicity [4]. Continued over-the-counter availability and limited pharmacovigilance awareness in permissive countries may contribute to underrecognition and delayed diagnosis of metamizole-induced agranulocytosis [2,5].

Estimates of the incidence of metamizole-induced agranulocytosis range from 0.5 to 1.6 cases per million person-days of exposure, although underreporting remains a concern in countries with limited pharmacovigilance [6,7]. Reported case fatality rates range from 10% to 20%, particularly in older adults or those with delayed diagnosis [8].

Diagnosis is often delayed due to nonspecific symptoms and overlapping antimicrobial use, which may mask the hematologic toxicity and mimic infectious syndromes. While previous case reports have described similar presentations [3,5], this case adds to the literature by illustrating the diagnostic challenges posed by concomitant infection and antimicrobial therapy.

We present a case of severe neutropenia caused by metamizole exposure, aiming to raise awareness of this adverse event and emphasize the importance of early detection and prompt drug withdrawal. Although other potential causes of neutropenia may coexist, the temporal relationship and clinical findings support a drug-induced etiology. This case further illustrates the paradox between the widespread, often unsupervised, use of metamizole and the rarity with which its potentially fatal hematologic complications are recognized.

## Case presentation

A 58-year-old male with a history of hypertension, type 2 diabetes, dyslipidemia, and obstructive sleep apnea underwent surgical repair following right shoulder trauma in June 2024. Postoperatively, he was prescribed oral metamizole 575 mg every eight hours for pain control. He returned to the emergency department days later due to wound dehiscence with purulent drainage. A computed tomography (CT) scan of the right shoulder revealed no evidence of fracture displacement or prosthetic failure, with the osteosynthesis material appearing adequately positioned. Mild glenohumeral misalignment, periarticular soft tissue densification, and small bone fragments in surrounding soft tissues were noted, but no signs of deep infection or osteomyelitis were identified. Based on these findings, the orthopedics team excluded surgical site infection. However, empirical antibiotic therapy was initiated with flucloxacillin, later switched to amoxicillin-clavulanate, to cover common skin flora in the context of superficial wound dehiscence. Due to clinical deterioration and the identification of multidrug-resistant *Pseudomonas aeruginosa *in wound exudate, antimicrobial therapy was escalated to meropenem and subsequently to ceftazidime-avibactam. Partial clinical improvement was achieved, and the patient was discharged on oral ciprofloxacin.

He was readmitted on July 30 with fever, absolute neutropenia, anemia, and elevated C-reactive protein. Ciprofloxacin was discontinued and meropenem reinitiated. Throughout August and September, he experienced multiple episodes of febrile neutropenia, requiring broad-spectrum antibiotics (piperacillin-tazobactam, meropenem, ceftazidime-avibactam), antifungal therapy (fluconazole), and granulocyte colony-stimulating factor (G-CSF).

Infectious workup was notable for a new isolation of multidrug-resistant *Pseudomonas aeruginosa* in ureteral exudate. Despite targeted antimicrobial therapy, the patient remained febrile and profoundly neutropenic. Imaging, viral serologies, and autoimmune markers were unremarkable (Table 1).

**Table 1:** Infectious workup. Most microbiologic and imaging findings were negative or non-specific, helping exclude infectious or malignant causes of neutropenia. SARS-CoV-2: severe acute respiratory syndrome coronavirus 2; RSV: respiratory syncytial virus; IGRA: interferon-gamma release assay; EBV: Epstein–Barr virus; CMV: cytomegalovirus; HIV: human immunodeficiency virus; CT: computed tomography

Test	Sample/Modality	Date	Result
Wound Culture	Post-operative wound pus	Jun 29	*Pseudomonas aeruginosa*, multidrug-resistant
Shoulder CT Scan	Right shoulder imaging	Jun 29	No signs of osteomyelitis or deep infection; mild glenohumeral misalignment and periarticular soft tissue densification with small bone fragments
Ureteral Exudate Culture	Ureteral drainage	Aug 27	*Pseudomonas aeruginosa*, multidrug-resistant
Blood Cultures	Peripheral blood	Aug 27	Negative
Urine Culture	Midstream urine	Aug 27	Negative
Respiratory Viral Panel	Nasopharyngeal swab	Aug 27	Negative for SARS-CoV-2, influenza A/B, RSV
Interferon-Gamma Release Assay (IGRA)	Blood	Aug 27	Negative
Galactomannan Antigen	Serum	Aug 27	Negative
Serologies	Serum	Aug 27	Negative for EBV, CMV, HIV, toxoplasmosis
Transthoracic Echocardiogram	Cardiac ultrasound	Aug 29	No signs suggestive of endocarditis
Chest CT Scan	Thoracic imaging	Aug 29	No parenchymal, pleural, or mediastinal changes; minor sequelae in the middle lobe

Persistent neutropenia prompted further evaluation, including assessment of nutritional parameters. All results were within normal limits (Table 2).

**Table 2:** Neutropenia workup. CD: cluster of differentiation; β2-microglobulin: Beta-2 microglobulin

Test	Sample/Modality	Date	Result	Reference Range
Reticulocytes	Blood	Aug 19	1.4%	0.5–2.5%
Folate			9.2 ng/mL	2.5–20 ng/mL
Vitamin B12			450 pg/mL	180–914 pg/mL
Iron			160 ng/mL	30–300 ng/mL
Abdominal Ultrasound	Imaging	Aug 22	No abnormalities or splenomegaly	—
Serum Protein Studies	Blood	Aug 28	Elevated immunoglobulins and light chains with normal ratio; β₂-microglobulin: 2.9 mg/L	β₂-microglobulin: <2.4 mg/L

**Figure 1:**
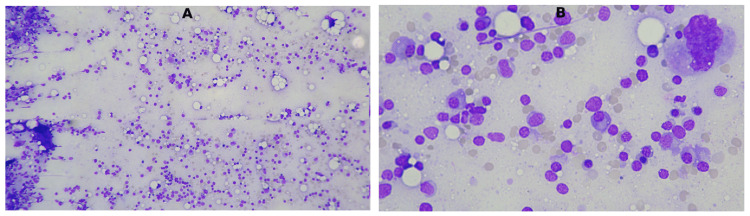
Bone marrow aspirate smear stained with May-Grünwald Giemsa. A: low-power field (10x) showing overall hypocellularity; B: higher magnification (40x) demonstrating absence of granulocytic precursors, with preservation of erythroid and lymphoid lineages. Findings are consistent with selective granulocytic suppression seen in drug-induced agranulocytosis.

A bone marrow aspirate was performed, revealing marked granulocytic depletion with relative preservation of erythroid and lymphoid precursors (Figure 1).

**Figure 2:**
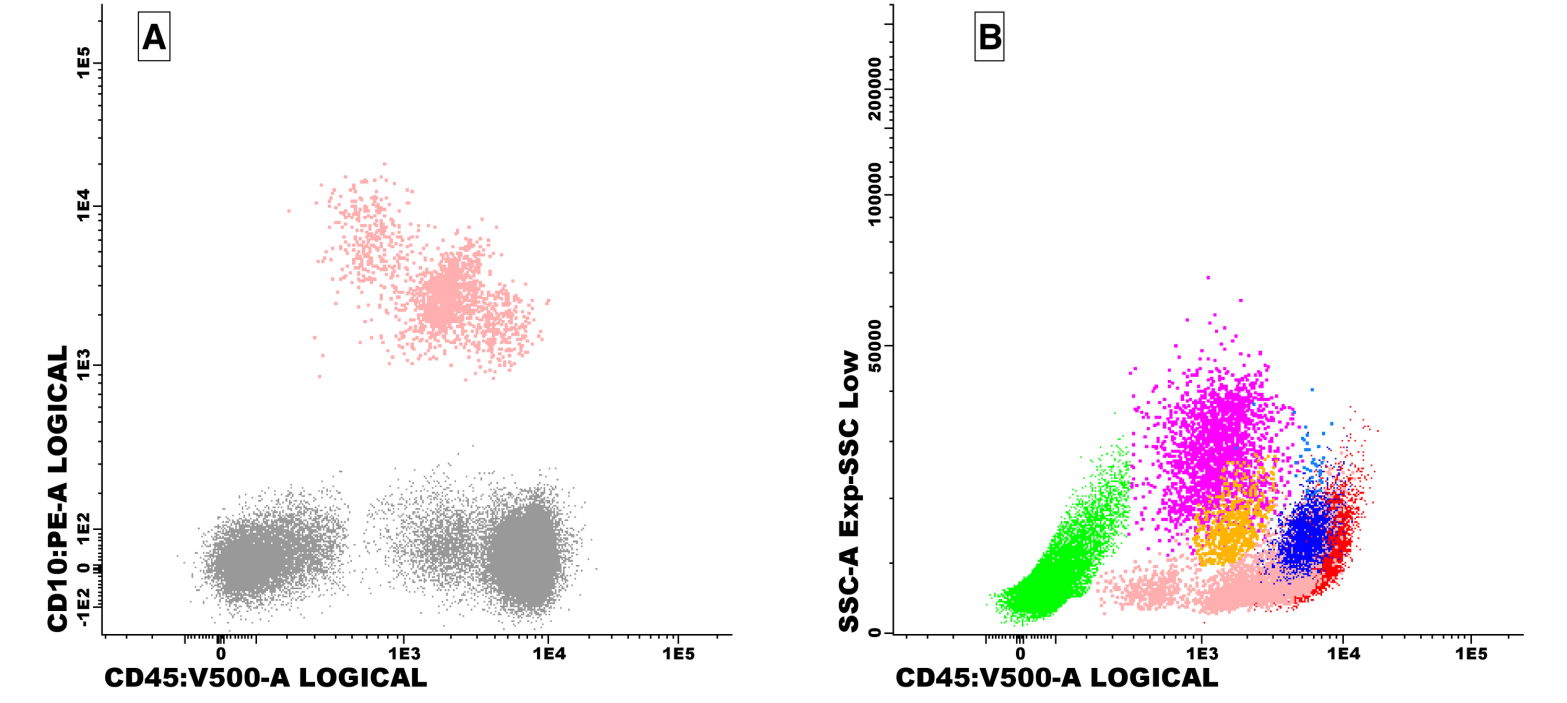
Bone marrow immunophenotyping. A: CD45 vs. CD10 dot plot showing no expansion of CD10⁺ precursor populations; B: CD45 vs. side scatter (SSC) plot demonstrating preserved hematopoietic lineages, including erythroblasts (green), B cells (pink), NK cells (dark blue), T cells (red), precursors (yellow), and monocytes (light blue). Findings support selective granulocytic suppression without evidence of clonal lymphoid expansion.

Bone marrow flow cytometry identified no clonal lymphoid populations. Immunophenotyping demonstrated mature B cells without light chain restriction, a small population of polyclonal plasma cells, and no expansion of CD10⁺ precursor cells (Figure 2).

**Figure 3:**
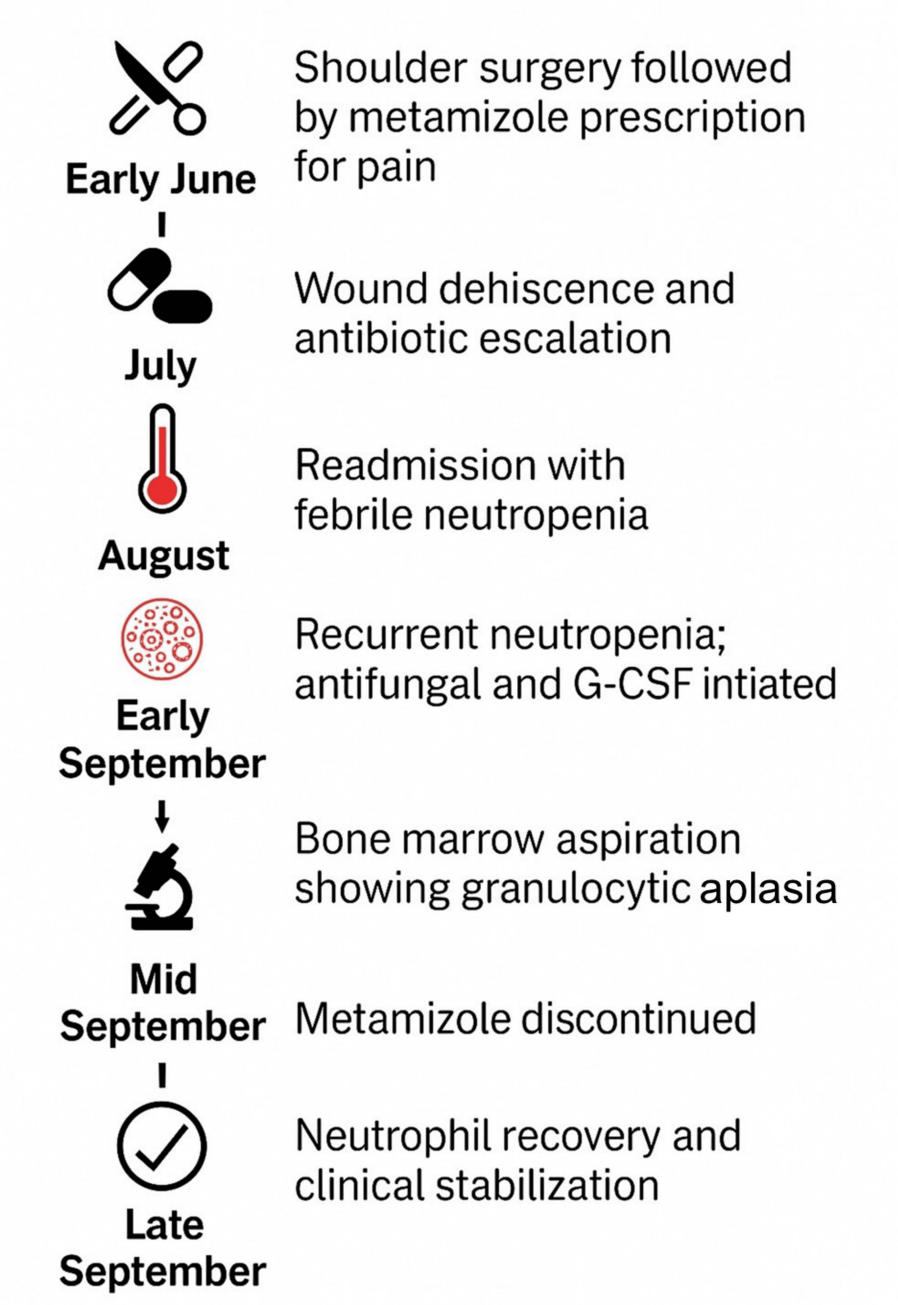
Timeline of clinical events and metamizole exposure. G-CSF: granulocyte colony-stimulating factor Figure created by the authors of the study. Icons obtained from Flaticon (Freepik Company, S.L., Spain) under a free license.​

**Figure 4:**
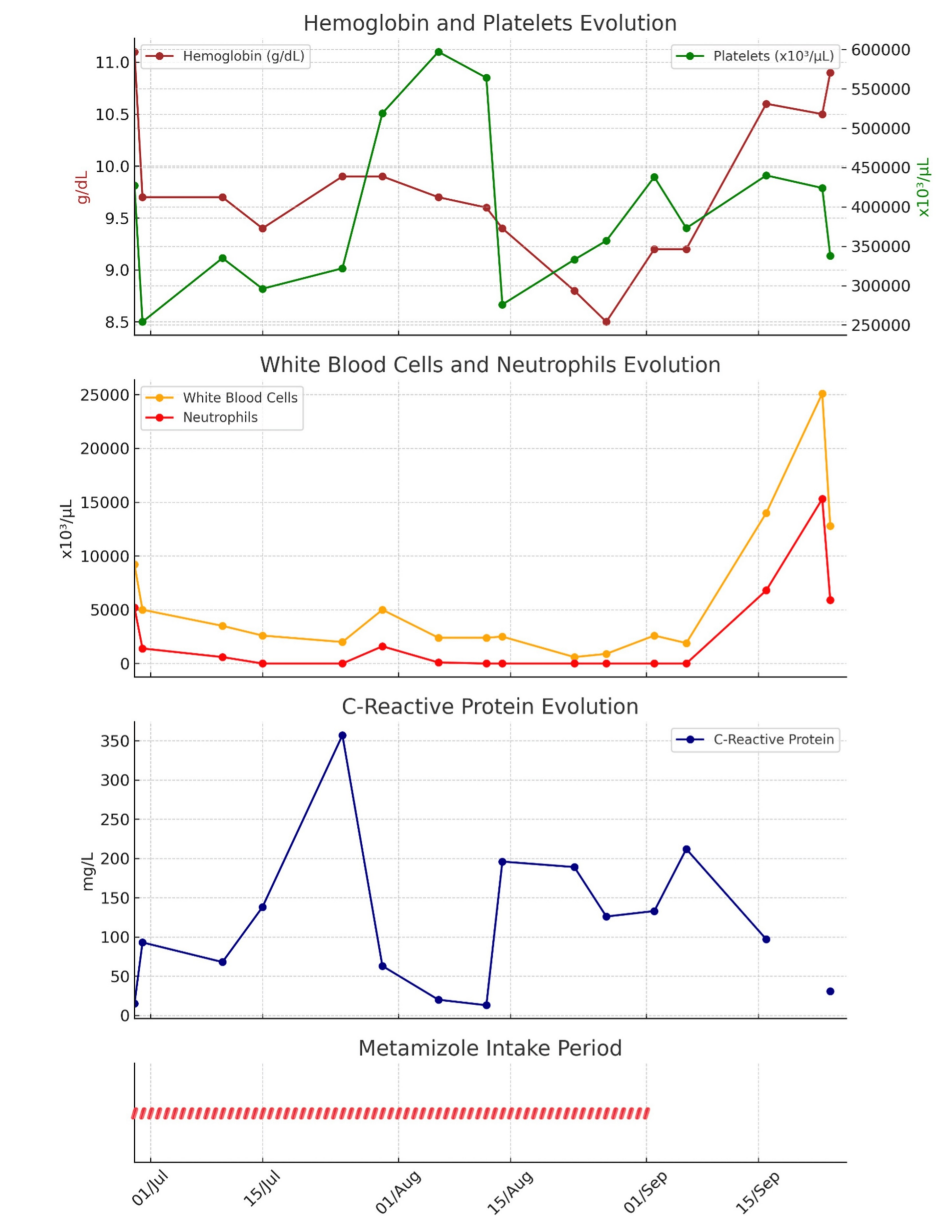
Laboratory trend during hospitalization. Laboratory trend during hospitalization, showing the evolution of hemoglobin and platelets, white blood cells and neutrophils, and C-reactive protein (CRP). The bottom panel illustrates the metamizole intake period, marked by red capsule icons, from June 29 to September 1, 2024. The timing aligns with the onset of hematologic toxicity and subsequent recovery.

Considering the inconclusive etiology and treatment failure, a detailed medication review was performed. Continuous metamizole use since June was identified. Its withdrawal in mid-September led to progressive hematologic recovery (Figures 3, 4), strongly supporting a diagnosis of metamizole-induced agranulocytosis.

## Discussion

Metamizole-induced agranulocytosis is an uncommon but potentially life-threatening adverse drug reaction. In our patient, the diagnosis was retrospectively confirmed based on temporal correlation, exclusion of infectious, autoimmune, or malignant causes, and complete hematologic recovery following drug discontinuation. The patient experienced full clinical resolution without recurrence after supportive care and G-CSF administration. Causality was further supported by a Naranjo Adverse Drug Reaction Probability Scale score of 7, indicating a “probable” relationship between metamizole and the observed agranulocytosis, with no plausible alternative causes identified.

Bone marrow aspirate smears (Figure 1) revealed overall hypocellularity and a selective absence of granulocytic precursors, with preserved erythroid and lymphoid lineages, a pattern typical of idiosyncratic toxic injury described in the literature [1-3,6].

Bone marrow immunophenotyping (Figure 2) demonstrated preserved distribution of hematopoietic lineages, including erythroblasts, B cells, T cells, NK cells, and monocytes, without evidence of clonal lymphoid expansion. Together, these findings are consistent with selective granulocytic suppression in drug-induced agranulocytosis.

Although rare, population-based studies suggest that the incidence of metamizole-induced agranulocytosis ranges from approximately 0.5 to 1.6 cases per million person-days of exposure [7]. A large retrospective matched cohort study in Germany reported a 7.6-fold increase in the risk of agranulocytosis among metamizole users compared to non-users, with the risk being particularly elevated within the first two weeks of treatment [8]. In Latin America, where metamizole is commonly used, recent observational data have documented several fatal outcomes, with reported case fatality rates reaching 20%, especially in older adults or those with delayed diagnosis [9]. These findings emphasize the need for heightened clinical vigilance and rapid drug discontinuation when hematologic toxicity is suspected.

Diagnosis was delayed by nonspecific clinical features and overlapping antimicrobial use, an issue well documented in the literature, as agranulocytosis often presents insidiously and mimics infectious syndromes. A report by Fernandes et al. [5] further illustrates this risk, describing a recurrence of agranulocytosis following inadvertent re-exposure to metamizole. These findings emphasize the importance of maintaining a high index of suspicion, especially in patients with prolonged febrile syndromes and unexplained cytopenias.

Regulatory differences across countries highlight a paradox: while metamizole remains widely available with limited monitoring in countries like Portugal and Spain, it is banned or restricted in others (e.g., the United States, United Kingdom, and Nordic countries) due to hematologic safety concerns [2,4,5].

While our report contributes to this body of evidence, several limitations must be acknowledged. First, causality was established through clinical judgment and exclusion of alternative causes, without confirmatory tests such as drug rechallenge or lymphocyte stimulation. Second, pharmacogenetic testing was not performed, limiting insight into individual susceptibility. Third, the single-center nature of this report may limit generalizability to broader prescribing contexts. Nonetheless, the strength of the temporal association, the consistency of findings with previously reported cases, and the hematologic recovery following drug withdrawal provide compelling evidence of causality.

In this context, genetic predisposition has emerged as a promising area of investigation. Although pharmacogenetic testing was not performed in this case, genome-wide studies have identified associations between metamizole-induced agranulocytosis and polymorphisms in HLA-B*27:05, NAT2, and cytochrome P450 isoenzymes [6]. While these findings remain within the research domain and are not yet integrated into standard care, they may help explain interindividual and interethnic differences in susceptibility, ultimately informing future strategies for risk stratification.

Given the ongoing availability of metamizole in many countries and its frequent use in treating febrile syndromes, clinicians should maintain a high level of vigilance when managing patients with unexplained neutropenia. Thorough medication reconciliation, including non-prescription drugs, is essential, especially in settings where over-the-counter access persists. Broader public health strategies, such as electronic prescribing alerts, mandatory adverse event reporting, and clinician-targeted education campaigns, are essential to mitigate the risk of preventable hematologic toxicity associated with metamizole.

## Conclusions

This case reinforces the need to consider metamizole as a potential cause of febrile neutropenia. Early recognition and prompt drug withdrawal are critical to improving patient outcomes. Furthermore, the continued over-the-counter availability of metamizole in several countries underscores the urgent need for strengthened regulatory surveillance and public awareness campaigns to prevent avoidable hematologic complications.
